# Polybrominated Diphenyl Ethers (PBDEs) in a Large, Highly Polluted Freshwater Lake, China: Occurrence, Fate, and Risk Assessment

**DOI:** 10.3390/ijerph15071529

**Published:** 2018-07-19

**Authors:** Jianchao Liu, Guanghua Lu, Fuhai Zhang, Matthew Nkoom, Zhenhua Yan, Donghai Wu

**Affiliations:** 1 Key Laboratory of Integrated Regulation and Resources Development, College of Environment, Hohai University, Nanjing 210098, China; jianchao-liu@hhu.edu.cn (J.L.); matthewnkoom@gmail.com (M.N.); hwahuer@hhu.edu.cn (Z.Y.); wdh1018@hhu.edu.cn (D.W.); 2 Water Conservancy Project & Civil Engineering College, Tibet Agriculture & Animal Husbandry University, Linzhi 860000, China; 3 Anhui Environmental Monitoring Center, Hefei 230061, China

**Keywords:** PBDEs, distribution, bioaccumulation, risk assessment, Chaohu Lake

## Abstract

Polybrominated diphenyl ethers (PBDEs) were extensively investigated in water, sediment, and biota samples collected from Chaohu Lake basin in China. The total concentrations of eight PBDEs (Σ_8_PBDEs) were in the ranges of 0.11–4.48 ng/L, 0.06–5.41 ng/g, and 0.02–1.50 ng/g dry weight (dw) in the water, sediment, and biota samples, respectively. The concentrations showed wide variations in the monitoring area, while the congener profiles in all the water, sediment, and biota samples were generally characterized by only a few compounds, such as BDE-47, BDE-99, and/or BDE-209. The spatial analysis depicted a decreasing trend of PBDEs from west to east Chaohu Lake, consistent with regional industrialization degree. The distributions of PBDE congeners in the biota samples were similar to the compositional profiles in the water, which were dominated by BDE-47 and/or BDE-99. Nevertheless, BDE-47 and BDE-153 in the brain tissue showed a higher accumulative potential than PBDEs in other tissues as well as the whole body, with 96% relative contribution of Σ_8_PBDEs. The noncarcinogenic risk values estimated for BDE-47, BDE-99, and BDE-153 indicated that the specific risk associated with the studied water and foodstuffs is limited. However, there is a potential mixture ecotoxicity at three trophic levels at some sampling points in the water, which should draw considerable attention.

## 1. Introduction

Polybrominated diphenyl ethers (PBDEs) as a type of brominated flame retardant have been a major contaminant in aquatic ecosystems [1], and are constituents of three commercial mixtures: pentabromodiphenyl ether (penta-BDE), octabromodiphenyl ether (octa-BDE), and decabromodiphenyl ether (deca-BDE) [2]. Because of their ubiquitous presence, bioaccumulation, and toxicity, penta- and octa-BDE mixtures have been restricted in some regions of the world, such as the European Union and parts of the United States [3]. However, deca-BDE mixture continues to be extensively used in many countries, including China [4]. Deca-BDE has a tendency to break down into more bioavailable lower-brominated congeners in the environment [5]. For instance, BDE-209, the main component of deca-BDE commercial mixture, can degrade or metabolize into lower-brominated congeners (e.g., BDE-47, BDE-99, and BDE-100) [6,7]. Low- and high-brominated congeners have been widely detected in environmental and biological samples, including air [8], water [9], sediments [10,11], soil [12], plant, aquatic organism [13], marine mammal [14], bird [15], and human tissues [16], collected from all over the world. Such contaminant levels may threaten human health, especially for local inhabitants. Thus, it is of great significance to continuously study the current contamination level of PBDEs in the various environmental media [17].

Chaohu Lake, a typical shallow lake, is one of the five largest freshwater lakes in China with a mean depth of 2.69 m and a surface area of 770 km^2^ [18]. The lake (E117°16′54″~E117°51′46″, N31°25′28″~N31°43′28″) is situated on flood plains between the Yangtze River and the Huaihe River. More than 9.1 million people live in the Chaohu Lake basin. In the whole basin of Chaohu Lake, there are 33 rivers connected to the lake. More than 90% of the total inflow is supplied by nine major inflowing rivers, including Hangbu River, Fengle River, Nanfei River, Baishishan River, Pai River, Shiwuli River, Zhao River, Zhegao River, and Shuangqiao River. Among them, Hangbu River is the largest river in Chaohu Lake basin by volume and contributes to 65% of the total inflow. The Yuxi River is the only outflowing river linking Chaohu Lake and Yangtze River. As a flowing lake, the riverine runoff is considered to be one of the important routes for the transport of contaminants to Chaohu Lake. Because of the increasing human activities in the lake basin in recent decades, Chaohu Lake has suffered from serious pollution and eutrophication. Recently, various classes of contaminants have been detected in the water, suspended particulate matter (SPM), and sediment of the lake [19]. However, few studies have focused on the comprehensive investigation on PBDEs in Chaohu Lake basin, including Chaohu Lake, the outflowing and inflowing rivers, and sewage treatment plant effluents, especially for the bioaccumulation and human health risk of PBDEs in this area.

Pollutants can be accumulated in aquatic organisms by direct absorption from the water or uptake through diet. Aquatic organisms at high trophic levels, such as fish, are likely to be the particularly vulnerable because of limited reproduction rates. In addition, humans, at the top of the food chain, are likely to ingest these compounds by consumption of aquatic products [20]. Any data on levels and distribution of PBDEs in the environmental media, especially fish, are of high significance not only for assessing the state of the ecological risk but for providing new health perspectives. This study therefore aimed to (1) investigate the contamination of eight PBDEs in the water and sediment from the major inflowing and outflowing rivers, large sewage treatment plants (STPs), and Chaohu Lake in order to track their temporal and spatial occurrence and potential sources; (2) determine the PBDE concentration in fish to elucidate the uptake potency, tissue distribution, and biological risk of PBDEs residues in wild fish; and (3) evaluate the potential health risk based on the bioaccessibility of PBDEs in the raw water and fish.

## 2. Materials and Methods 

### 2.1. Materials

Standards of the individual target analytes, including BDE-28, BDE-47, BDE-99, BDE-100, BDE-153, BDE-154, BDE-183, and BDE-209 as well as the internal standard, ^13^C-BDE-153, were purchased from AccuStandard (New Haven, CT, USA). Silica gel, acetone, n-hexane, dichloromethane, ethyl acetate, and methanol were obtained from Merck Serono Co., Ltd. (Darmstadt Germany). Water was purified using a Milli-Q integral water purification system (Millipore, Milford, MA, USA).

### 2.2. Study Area and Sample Collection

Water samples from eight major inflowing rivers of Chaohu Lake (P1~P8, about 2 km upstream of the estuaries) and one outflowing river (P9 in Yuxi River) were obtained in June and October. In addition, water samples from eight estuaries (H1~H8), four lakeside towns (H9~H12), and the centers of western Chaohu Lake and eastern Chaohu Lake were collected in in June and October, to observe the temporal and spatial variation of the PBDEs in the lake. P1~P9 and H1~H8 were also the key control sections of the Ministry of Environmental Protection of the People’s Republic of China (PRC) in Chaohu basin. The 24 h composite effluent samples from five main STPs (the final effluents of W1, W2, and W3~W5 were discharged into the Pai River, Shiwuli River, and Nanfei River, respectively) were collected in October to evaluate the potential source of the PBDEs in Chaohu Lake. In all the sample points, triplicate water samples were collected sequentially in dark amber glass bottles, according to the technical specification requirements for monitoring of surface water and wastewater. Samples were transported to the laboratory in a cooler and filtered through 0.45 μm prewashed glass fiber filters. The samples were then stored in the dark at 4 °C until the solid-phase extraction (within 48 h) and analyzed over a period of 4 days.

**Figure 1:**
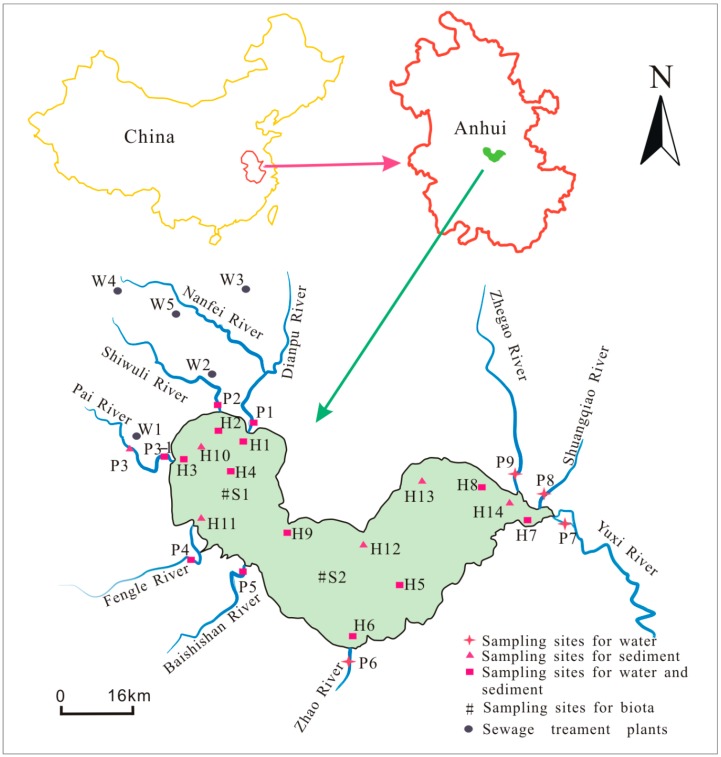
Maps of the sampling sites in Chaohu Lake basin, China.

A total of 20 districts were selected as surface sediment sampling spots in October. Simultaneously, sludge samples from an STP (W1) were collected to analyze PBDE transport in the process of sewage treatment. The top 5 cm layer of sediments collected using a stainless steel grab sampler was scooped using a precleaned stainless steel scoop. The collected sediment samples were wrapped in tinfoil and enclosed in ziplock bags to avoid pollutant losses, and placed in a sample box with Drikold. After transporting to the laboratory, sediment samples were stored at −20 °C. Fish were captured with bottom trawling in October in two sampling points of S1 and S2, which were located in the centers of the western and eastern parts of the lake, respectively. After capture, fish samples were immediately anesthetized by immersion in tricaine methanesulfonate and transported on drikold to the laboratory for further pretreatment. In the laboratory, the whole fish, fish tissues (liver, brain, gill, and muscle), and sediments were each lyophilized at −60 °C and held at −80 °C until processing. The detailed locations of the sampling sites are displayed in Figure 1. 

### 2.3. Sample Extraction and Analyses

Detailed information on sample extraction and instrumental analysis is given in the Supplementary Information (SI). Briefly, water samples were filtered through 0.45 µm glass fiber filters (GF/F, Whatman, Clifton, NJ, USA) to remove particles. The filtered water samples mixed with 10% volumes of methanol were extracted by solid-phase extraction (SPE) on Hydrophilic-Lipophilic Balance cartridges (200 mg, Waters, Milford, MA, USA), followed by elution with ethyl acetate and dichloromethane. PBDEs in the sediment and biota samples were extracted by n-hexane/dichloromethane (4:1) using an ASE 300 pressurized liquid extraction system (Dionex, Sunnyvale, CA, USA), and synchronously purified using anhydrous sodium sulfate and activated Florisil. PBDEs were measured on a Bruker450 GC coupled to a Bruker320 series Triple Quadrupole mass spectrometer (Fremont, CA, USA) equipped with a DB-5HT capillary column (15 cm × 250 μm × 0.1 μm). The optimized MS/MS parameters of eight PBDEs are presented in Table S1.

### 2.4. Quality Assurance and Quality Control

All data were subjected to strict quality control procedures. The target compounds in the samples were identified by comparing their retention times with those of native standards (within ± 0.01 min) and confirmed by comparing the peak area ratio of the two product ions in the samples with native standards (accepted when within ±20%). Two procedural blanks were determined simultaneously for each set of the ten samples by going through the same extraction and cleanup procedures, and concentrations of most target compounds were below the detection limit. The average amount of the blanks was subtracted from the amount in the samples. Recoveries were evaluated by spiking blanks with low (5 ng/L), medium (20 ng/L), and high (50 ng/L) concentrations, the standard for the water samples, ranged from 75% to 110%. Recoveries of the target compounds in the sediment and biota samples were obtained in the range of 65%~110%. However, BDE-209 has low method recoveries due to its large molecular weight, high adsorptivity, and low sensitivity. The recovery rates were from 50% to 80% and from 65% to 110% for the water samples and sediment/biota samples, respectively (Tables S2 and S3).

The Instrument Detection Limit (IDL) is the concentration equivalent to 3 times the signal-to-noise ratio (S/N); this ranged from 0.015 to 0.15 pg. Method detection limits (MDLs) were calculated from the standard deviation (SD) of seven replicate injections of fortified matrix extracts. The MDLs ranged from 0.014 to 0.6 ng/L and 4 to 20 pg/g for the water and sediment/biota samples, respectively (Table S4). Good linearity was observed over the specified concentration range (1–100 μg/L) with correlation coefficients all higher than 0.997 (Table S5). The precision of the method was evaluated in terms of its repeatability calculated as the percentage relative standard deviation (RSD, %) of the recovery rates. The precision of the method evaluated in terms of six replicates of spiked matrix samples was 1.4%~6.0% of RSD at 20 ng/L for the water samples and 3.4%~12.8% of RSD at 5 ng/g for the sediment/biota samples, respectively. The accuracy, exactitude, and sensitivity of this method achieve the required degree of the USEPA1614 method. If the concentration of an individual congener was below the MDL, its concentration was assumed to be MDL/2 for statistical analysis.

### 2.5. Parameter Measurement and Statistical Analysis

The noncancer risk associated with intake of PBDEs for people was assessed using previously reported methods. Ecotoxicity of the target compounds in the water was assessed using the risk quotient (RQ) and mixture risk quotient (MRQ) on nontarget organisms. Detailed calculation methods are described in SI. SigmaPlot 12.5 was used to perform the regression analysis on MRQ_STU_ versus MRQ_MEC/PNEC_ (STU, MEC and PNEC represent the sum of toxic units, measured environmental concentration, and predicted no effect concentration, respectively). A *p*-value of 0.05 was used for the test significance.

## 3. Results and Discussion

### 3.1. Occurrence of PBDEs in Water Collected from Chaohu Lake Basin

**Figure 2:**
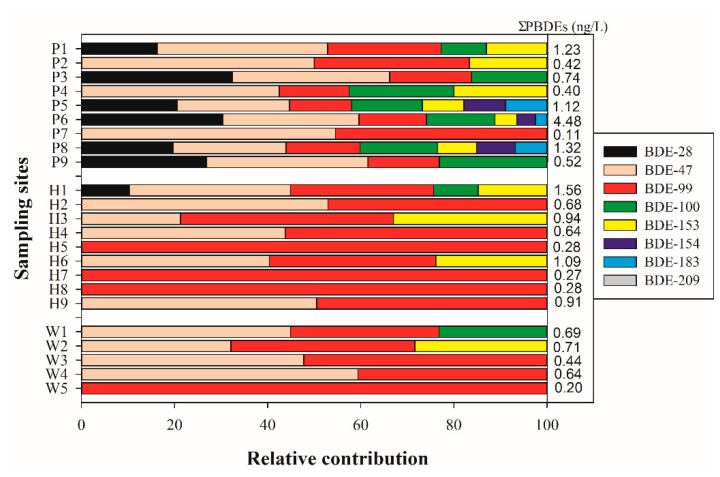
Compositional pattern of polybrominated diphenyl ethers (PBDEs) in water samples collected from rivers (P1–P9), Chaohu Lake (H1–H9), and sewage treatment plant (STP) effluents (W1–W5).

Seven of the eight PBDEs were detected in at least three rivers in Chaohu Lake basin. Details of the change of PBDE concentrations in June and October can be found in the SI (Table S6). Overall, the pollution levels of PBDEs in October were 1–2 times higher than those in June; only the data from October was included in Figure 2. In all the rivers connected to Chaohu Lake (sampling sites P1–P9), BDE-47 and BDE-99 were the most frequently detected compounds, followed by BDE-28, BDE-100, and BDE-153, whereas BDE-154 and BDE-183 were only sporadically detected (Figure 2). Among all the selected PBDEs, BDE-47 and BDE-99 were also the predominant PBDEs, with relative contributions ranging from 37.5% to 100%.

Eight major inflowing rivers showed different spatial distributions of PBDEs. In general, the Zhao River (P6) had the highest mean concentrations of the eight PBDEs (Σ_8_PBDEs = 4.48 ng/L), followed by Shuangqiao River (1.32 ng/L), Nanfei River (1.23 ng/L) and Baishishan River (1.12 ng/L), and Pai River (0.74 ng/L), while Zhegao River, Shiwuli River, and Fengle River presented quite similar levels of pollution with concentrations of Σ_8_PBDEs ranging from 0.40 to 0.52 ng/L. However, monomer species, individual concentrations, and Σ_8_PBDEs in the outflowing river (Yuxi River) were obviously lower than those in the inflowing rivers.

There is only one study about PBDEs performed on the inflowing rivers of Chaohu Lake. Wang et al. investigated the contamination of PBDEs in seven inflowing rivers of the Chaohu Lake and found out that the Σ_8_PBDEs varied from 0.31 to 84 ng/L, with the individual concentrations of BDE-47, BDE-99, and BDE-153 being <0.012–0.36, <0.012–1.3, and <0.012–0.77 ng/L, respectively [21]. In our study, BDE-47, BDE-99, and BDE-153 concentrations ranged from 0.17 to 1.31, from 0.06 to 0.65, and from <0.03 to 0.21 ng/L, respectively, which was comparable to the research. 

BDE congener contributions relative to the total concentrations in the effluent samples from the five STPs are presented in Figure 2 (W1–W5). The Σ_8_PBDEs in the effluent ranged from 0.2 to 0.81 ng/L in the five STPs. The major congeners of PBDEs detected in the effluent were BDE-47 and BDE-99, with relative contributions ranging from 71.6% to 100%. In addition, BDE-100 and BDE-153 were also detected in the effluent samples of W1 and W2, respectively, with mean concentrations of 0.16 and 0.23 ng/L (Table S7). To assess the possible contribution of the STP (W4 in our study) to the Nanfei River, a previous study found that the mean concentration of Σ_7_PBDEs (excluding BDE-209) was only 0.08 ng/L, which was lower than that in the upstream site of Nanfei River [22]. In our study, as compared to the pollution situation of the inflowing river, monomer species, the concentrations of PBDE congeners, and Σ_8_PBDEs in the effluents of five STPs were significantly reduced. This indicates that the domestic effluents have not been a primary source of lower-brominated BDE congeners in the inflowing rivers of Chaohu Lake basin.

**Figure 3:**
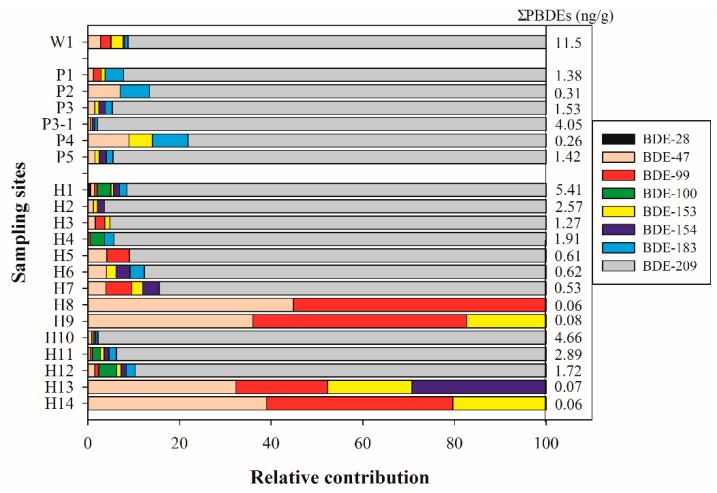
Compositional pattern of PBDEs in sediment/sludge samples collected from rivers (P1–P5), Chaohu Lake (H1–H14), and STP effluents (W1).

In this study, BDE-209 was below the 0.6 ng/L MDL in the effluent of the STPs; however, it is the most abundant congener in the sludge with >91% of BDE-209 partitions into the sludge (Figure 3). Three other large contributors to the sludge are BDE-47, BDE-99, and BDE-153, which was similar to the traditional distribution pattern. It has been previously shown that the sum of the major congeners in the penta-formulation (BDE-47, 99, 100, 153, and 154) comprises 88% of the total PBDEs in the effluent, while BDE-209 is only 6%; however, in sediment BDE-209 was more than 99% of relative contributions due to its strong absorption [23]. Wang et al. found that PBDEs were eliminated mainly by sedimentation processes, and the removal rates of PBDEs increased with the increase of their solid–water partitioning coefficients, such as BDE-209 (log Kow ≈ 10) [22]. Thus, further research is important on the effects of primary sludge and waste-activated sludge in the STP on the fate, transport, and resulting environmental loadings of PBDEs. 

Water samples from all of the nine sites (H1–H9) contained detectable concentrations of PBDEs, indicating that these contaminants are widely spread in Chaohu Lake. The concentrations of PBDEs determined at each site for individual congeners are shown in Table S8. The Σ_8_PBDEs ranged from 0.16 to 1.02 ng/L in June and 0.27 to 1.56 ng/L in October, which was lower than those in the rivers connected to Chaohu Lake. Overall, the higher Σ_8_PBDEs for all of the sampling sites in Chaohu Lake were detected in the western part of Chaohu Lake located in an urban area with a mean concentration of 0.96 ng/L (H1–H4), followed by the coast of the eastern part of Chaohu Lake located in suburban areas with a mean concentration of 0.76 ng/L (H6, H8, and H9). The lowest total concentrations were found at the center of the east side of the lake (0.28 ng/L at site H5) and downstream of Chaohu Lake (0.27 ng/L at site H7). Figure 2 (H1–H9) shows the pattern of individual PBDE congeners of Chaohu Lake. Similar to water samples from the rivers and STPs, BDE-47 and BDE-99 were the dominant congeners in the surface water of Chaohu Lake, with concentrations of 0.10–0.54 and 0.16–0.48 ng/L, respectively. BDE-154, BDE-183, and BDE-209 were below the MDLs in all the sampling sites. These results indicate that the inflowing rivers are primary sources of PBDEs in Chaohu Lake and the PBDE contents may decrease because of the lake water dilution.

The concentrations of PBDEs in the surface water were usually below 1 ng/L, due to their inherently hydrophobic property. In China, the occurrence of PBDEs in surface water has been investigated in the three major river systems Pearl River, Yellow River, and Yangtze River at mean concentrations of Σ_7_PBDE (excluding 209) up to 0.08 ng/L [24], 1.26 ng/L [25], and 6.44 ng/L [26], respectively. The concentrations of PBDEs in Chaohu Lake basin were situated between the above concentrations, but were lower than those in the water of e-waste dismantling areas [27]. In addition, suspended particulate matter (SPM) samples collected from Chaohu Lake were investigated by He et al. [28]. They found that the concentration of Σ_7_PBDE and the concentration of BDE-209 ranged from 3.2 to 1124.7 ng/g (dw) and from 12.6 to 125.6 ng/g (dw) in the SPM, which were 2–3 orders of magnitude higher than those found in the sediments [28]. These results indicate that SPM may be a very important sink, and, further, a carrier of PBDEs in the aquatic systems.

### 3.2. The Occurrence of PBDEs in Sediment Collected from Chaohu Lake Basin

The occurrences of PBDEs in the sediment sampling sites of Chaohu Lake basin are shown in Tables S9 and S10 and summarized in Figure 3. Among the studied compounds, BDE-47 and BDE-209 were the most ubiquitous PBDEs detected in sediment samples from the rivers and Chaohu Lake, ranging from ND to 0.043 ng/g and ND to 4.95 ng/g, respectively. Unlike the water samples, the congener profiles were dominated by BDE-209, with average values accounting for 90.6% and 92.6% of the total PBDEs in the sediment samples of the rivers and Chaohu Lake, respectively. BDE-153, BDE-154, and BDE-183 were detected in most of the sediment samples, with concentrations ranging from 0.012 to 0.086 ng/g. BDE-99 and BDE-100 were detected in few sediment samples, at concentrations from 0.01 to 0.16 ng/g. BDE-28 was not detected in the sediments of the rivers, and it was only found in H1 of Chaohu Lake.

The mean concentration of the Σ_8_PBDEs ranged from 0.26 to 4.05 ng/g in the five inflowing rivers. The Paihe River presented the highest PBDEs levels, with Σ_8_PBDEs ranging from 1.53 (site P3) to 4.05 ng/g (site P3-1), followed by Baishishan River (site P5), Nanfei River (site P1), Shiwuli River (site P2), and Fengle River (site P4). Two sediment samples of P3 and P3-1 which were collected just before and after the outlets of W1 STP also presented the highest BDE-209 levels, ranging from 1.45 to 3.96 ng/g. The W1 STP receives wastewater mostly from residential regions and industry from the economic development zone of Hefei and Feixi. The discharge of the STP into Paihe River was approximately 0.3 million m^3^/day, which can contribute up to 75% of the Paihe River streamflow. In addition, several drain outlets were found from sampling sites P3 to P3-1, and effluent from the pond and village area were discharged into the estuary without any purification treatment, which may be the main cause of worsening water quality. 

As shown in Table S10 and Figure 3, four PBDEs (BDE-47, BDE-99, BDE-153, and BDE-209) were widely detected in the surface sediment of Chaohu Lake, with respective detection rates of 92.9%, 78.6%, 78.6%, and 71.4%. The mean concentration of Σ_8_PBDEs ranged from 0.06 to 5.41 ng/g at all the sampling sites, whereas H8, H9, H13, and H14 presented significantly lower levels (0.06–0.08 ng/g) than other sites, followed by H5, H6, and H7 (0.53–0.62 ng/g). Seven sampling sites are higher than 1 ng/g, which are almost located in the western region of Chaohu Lake. The highest and lowest concentrations of Σ_8_PBDEs were found at H1 and H8/H14, respectively. H1 is located at the estuary of Nanfei River which passes through Hefei city. H8/H14 is located at the mouth of Yuxi River which is the only outlet of the lake. In addition, in the North of Chaohu Lake (H8, H9, H13, and H14 sites), the sediment was predominately sludge dredged. The mean concentration of Σ_8_PBDEs from the western region (H1–H4, H10, and H11) of 3.12 ng/g was about 6.5 times higher than that obtained in the eastern region (H5–H9 and H12–H14) of 0.47 ng/g. This indicates that the contamination by PBDEs in the western region of Chaohu Lake is more serious than that in the eastern region. On the whole, the concentration of Σ_8_PBDEs and BDE-209 in the sediment from the western to eastern region decreased gradually, while relative contributions of other PBDE congeners (excluding BDE-209) increased gradually. It has been reported that BDE-209 can undergo photolytic debromination and metabolic degradation to lower-brominated BDE congeners (e.g., Tetra-BDE, Penta-BDE, and Hexa-BDE) [29]. Thus, it was possible for BDE-209 to be transformed into less-brominated congeners, such as BDE-47 and BDE-99, by natural sunlight, bacteria, and/or metabolism [24].

Previous studies revealed that riverine discharge was the main input pathway of PBDEs, PCBs, and PAHs into the lake [30]. The difference with respect to pollution degree in Chaohu Lake could be explained by the GDPs and populations of Hefei city and Chaohu city, which are located in the northwestern and southeastern shore of Chaohu Lake, respectively. For instance, in 2016, the urban population of ~5.5 million and GDP of ~620 billion RMB in Hefei city were about 7 times and 23 times those in Chaohu city, respectively. As a result, urban sewage and industrial wastewater from the western land of Chaohu Lake were deduced as the major contributors to the pollution in the aquatic ecosystem of Chaohu Lake. On the other hand, the eastern region of Chaohu Lake and Yangtze River are connected by Yuxi River. In wet periods, Chaohu Lake discharges water to the Yangtze River, while water from the Yangtze River is introduced into the eastern region of Chaohu Lake through Yuxi River in dry periods. Water transfer had positive effects on decreasing the concentrations of contaminants in the eastern region of Chaohu Lake [31].

The PBDE levels in Chaohu Lake were investigated four times in the past ten years. Due to the different targets of PBDE congeners in each study, only tworesearch papers with 8 BDE monomers as their target were collected. The total mean concentrations of PBDEs were 7.3 ng/g and 0.7 ng/g in the sediments samples collected from Chaohu Lake in 2006 [32] and 2009 [28], respectively. In our study, the mean concentration of PBDEs was 0.7 ng/g in the sediment of Chaohu Lake, which indicates that the pollution level of PBDEs has significantly reduced in the past ten years. The contamination status of PBDEs in sediment from Chaohu Lake was significantly lower than those found in the southeastern regions with rapid industrial development speeds and high economic levels (e.g., Guangdong, Jiangsu, and Zhejiang province in China) and similar to the values from Three Gorges Reservoir and Poyang Lake in China [33].

### 3.3. Bioaccumulation of PBDEs in Biota Samples Collected from Chaohu Lake 

**Figure 4:**
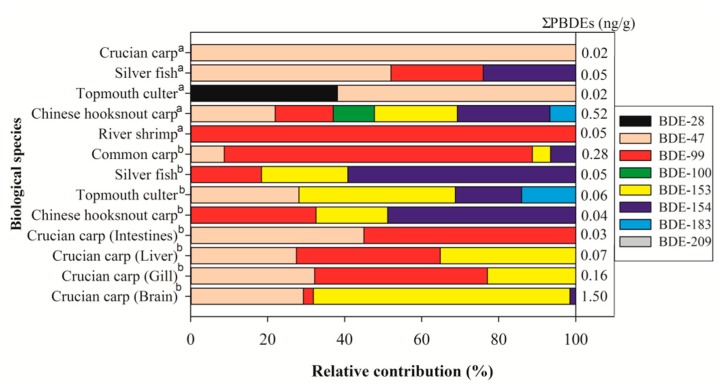
Congener pattern of PBDEs in aquatic species from Chaohu Lake (^a^ Biota samples collected from S1; ^b^ Biota samples collected from S2).

So far, bioaccumulation of PBDEs has not been monitored and reported in the whole body of fish collected from Chaohu Lake. To estimate the current status of PBDE contamination in biota, two sampling points (S1 and S2) were chosen in the centers of the western and eastern parts of the lake, respectively. Altogether, 58 samples of six edible fish species and two shrimp species were collected from the two sampling sites. The species collected from sampling Site S1 were crucian carp, silver fish, topmouth culter, Chinese hooksnout carp (*Opsariichthys bidens*), lake anchovy (*Coilia ectenes taihuensis*), river shrimp, and white shrimp (*Exopalaemon modestus*). From the center of the eastern part of the lake (S2), common carp, silver fish, topmouth culter, Chinese hooksnout carp, and lake anchovy were collected. PBDEs were not detected in the lake anchovy and white shrimp. In all of the samples detected, the Σ_8_PBDEs varied from 0.02 to 0.52 ng/g dw in the whole body of fish (Figure 4 and Table S11). The Σ_8_PBDEs in fish collected from the western part was found to be 0.132 ng/g, which is slightly higher than the value (0.108 ng/g) in fish from the eastern part.

Data on PBDE levels in fish from Chaohu Lake is scarce, but, overall, the levels observed in the present study were in line with or higher than those previously reported in 13 fish species collected from 11 coastal cities of Guangdong province (China) [34]. The relatively higher concentration of Σ_8_PBDEs was detected in Chinese hooksnout carp (S1 site) and common carp (S2 site), with mean concentrations of 0.52 and 0.28 ng/g, respectively. Chinese hooksnout carp are typically carnivorous fish and often feed on aquatic insects and tiddlers. The carnivorous fish generally occupy a higher trophic level of the food chain in an aquatic ecosystem, which may accumulate PBDEs when consuming other organisms contaminated with PBDEs. This trend is in agreement with findings from the whole-body measurement of dechlorane plus flame retardant in paradise fish (*Macropodus opercularis*), mosquito fish (*Gambusia affinis*), and Chinese hooksnout carp collected from an electronic waste recycling site, where Chinese hooksnout carp had the highest dechlorane plus (410 ng/g lipid weight) [35]. Chinese hooksnout carp is not an economically important species in Chaohu Lake, but it is a prey fish species for fish-eating birds, which may pose a threat to piscivorous avian species occupying high trophic levels.

The whole-body Σ_8_PBDEs for the bottom-dweller common carp were 4.7~7 times greater than those of the other species in the site S2, and BDE-99 was the most abundant congener (average proportion 80%). The higher concentrations observed in the common carp than in other species are in agreement with the higher levels found in bottom-dweller common carp and white sucker than other species in U.S. lakes [36] and the Canadian Great Lakes [37]. The higher PBDE levels in the common carp are likely due to their interactions with contaminated sediment in the areas. In addition, previous studies indicated that several factors such as species, trophic level, and possible biodegradation capacity may influence PBDE bioaccumulation in biota [38,39].

In order to understand the tissue distribution of PBDEs, we investigated the concentrations of PBDEs in the brain, gill, liver, intestines, and muscle tissues of crucian carp. In crucian carp, the brain showed highest total contaminant levels followed by the gill, liver, intestines, and muscle. Generally, highest Σ_8_PBDEs contents were detected in the brain tissue, suggesting that it is able to cross the blood–brain barrier and accumulate in the brain. This result implied that the brain may be a key target organ for PBDE toxicity and this may have direct effects on the nerve cells of organisms subjected to episodic or continual lifecycle exposure [40]. Pre- and/or post-natal exposure to PBDEs may cause long-lasting behavioral abnormalities, oxidative-stress-related damage, and interference with signal transduction and neurotransmitter systems [41]. In the brain, BDE-153 contributed to more than 65% of Σ_8_PBDEs, followed by BDE-47 (30%), which was similar to a previous study by Zhang et al. [42], where the BDE-153 in the brain was higher than in other tissues. Gills are the first organs in contact with polluted water and suspended sediment particles; therefore, they play an important role in contaminant uptake. This pattern was already seen for common carp in Negro River basin [43], where gills showed the highest total contaminant levels, followed by liver, gonads, and muscle (brain tissue was not measured). In the fish, liver levels provide an insight into the fresh contamination due to its role in detoxification and the biotransformation of xenobiotics. The levels of PBDEs in the liver were always higher than in the intestines in all the fish sampled. Among all the analyzed tissues, the muscle is of concern due to its implications for human consumption. However, in the muscle tissue, the PBDE levels were below the MDL.

### 3.4. PCA Analysis

**Figure 5:**
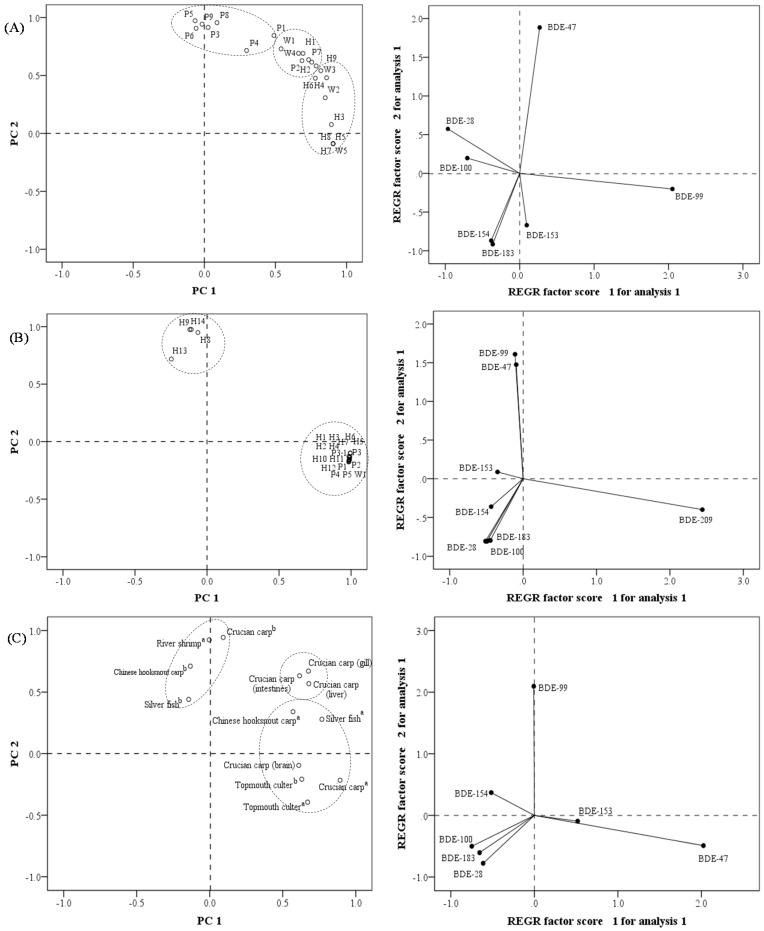
Principal component analysis (PCA) score plot and Regression (REGR) factor score plot for PBDEs in water (**A**), sediment (**B**), and biota (**C**) samples from Chaohu Lake basin (^a^ Biota samples collected from S1; ^b^ Biota samples collected from S2).

After normalizing by the total concentration, principal component analysis (PCA) was employed for further assessment of PBDEs in the water, sediment, and biota samples from the Chaohu Lake basins. As exhibited in Figure 5A, 87.4% of PBDE variation in the surface water of Chaohu Lake basin could be explained by PC1 (65.5%) and PC2 (21.9%) (eigenvalue > 1). It is distinctive that most of the samples from Chaohu Lake (H3–H9) and samples from the STPs (W2, W3, and W5) were characterized by PC1 with a signature of BDE-99. Also, the majority of samples from the connected rivers (P1, P3–P6, P8, and P9) had higher loading values of PC2 in the component plot and featured BDE-47. Fewer samples from the connected river (P2 and P7), Chaohu Lake (H1 and H2), and STPs (W1 and W4) were correlated positively with both PC1 and PC2. All the above results indicated the same source for the sampling group of inflowing rivers (P1, P3–P6, P8, and P9); for the sampling group of H1, H2, and P2; and for the sampling group of Chaohu Lake (H3–H9). Both BDE-47 and BDE-99 are the major congeners of the penta-formulation of PBDEs.

For the sediment of Chaohu Lake Basin (Figure 5B), 97.1% of the PBDE variation could be explained by PC1 (82.5%) and PC2 (14.6%) (eigenvalue > 1). All the samples had a similar contamination profile due to a high score in PC1 in the positive direction except for H8, H9, H13, and H14, manifesting the dominance of BDE-209 in most sites. The four outliers were all located in the north of Chaohu Lake, characterized by BDE-47 and BDE-99. As compared to other regions of Chaohu Lake, the north side has fewer inflowing rivers and just finished a dredging project; thus, there is less imported pollution. Considering that BDE-209 was still the dominant congener in these sediment samples, penta-BDE mixtures might be from degradation products of BDE-209. These degradation products could be released into the water from sediment and diffuse the entire lake through hydrodynamic action.

Regarding biota samples, the first two components (PC1, PC2) (eigenvalue > 1) could explain 35.5% and 32.6% of the total variance, respectively (Figure 5C). BDE-47 dominated all the fish samples collected from the S1 site (except for river shrimp), while BDE-99 was the main influencing factor for most fish collected from the S2 site (except for topmouth culter), which was similar to the spatial distribution of BDE-47 and BDE-99 in the water body of Chaohu Lake. BDE-99 is more likely to bioaccumulate in the river shrimp, while topmouth culter shows a high cumulative potential for BDE-47. Compared with the gill, liver, and intestines of crucian carp, which have similar bioaccumulation potential for BDE-47 and BDE-99, the brain is more susceptible to BDE-47 and BDE-153 accumulation.

### 3.5. Risk Assessment

#### 3.5.1. Health Risk Assessment

Potential human risks maybe exist due to the consumption of contaminated water and fish [44]. In Chaohu Lake, there is a thriving fishery and the estimated fish production reached 42,000 tons in 2014. The quality of fish is closely related to human health. The assessment of human risk via water and fish consumption from the lake is, therefore, an important issue.

The threshold values of Federal Environmental Quality Guidelines (FEQG) for PBDEs in Canada were used to estimate the PBDE concentrations: tri-BDEs (46 ng/L), tetra-BDEs (24 ng/L), and hexa-BDEs (120 ng/L) [45]. The tri-BDEs (ND-1.36 ng/L), tetra-BDEs (ND-1.31 ng/L), and hexa-BDEs (ND-0.39 ng/L) concentrations detected in this study were much lower than the threshold values of FEQG. These results indicated that the water quality in Chaohu Lake basin was safe according to the existing guidelines. To further estimate the risk of PBDEs in the water, the noncarcinogenic risk was calculated. Out of the eight PBDEs determined in this study, relevant toxicity data were available for only BDE-47, BDE-99, and BDE-153. Therefore, risk assessment could be carried out only for these three individual PBDE congeners. The estimated daily intakes (EDIs) of Σ_3_PBDEs for a normal exposure scenario (mean values) were 0.024 and 0.21 ng/kg body/day for adults via consumption of drinking water and fish per day, respectively (Table S12). Based on the average total EDI, the hazard quotient (HQ) values were calculated in order to estimate the noncancer risk for PBDE exposure (Table S12). As mentioned in Yu et al., body weight (BW) was assumed to be 63.1 kg and the fish consumption rate is 132 g/day for the average fisherman in China [46]. The reference dose (RfD) values from the draft U.S. Environmental Protection Agency Integrated Risk Information System (EPA IRIS) Toxicological Evaluations were 0.1 µg/kg-day for both BDE-47 and BDE-99, and 0.2 µg/kg-day for BDE-153 [47]. The total HQ values ranged from 4.1 × 10^−7^ to 7.2 × 10^−6^ and 4.1 × 10^−6^ to 4.2 × 10^−5^ for adults via consumption of drinking water and fish, respectively, and increased to 4.5 × 10^−6^ to 4.9 × 10^−5^ when eating and drinking happened simultaneously. All the HQ values were much lower than 1, suggesting that consumption of the water and fish in Chaohu Lake would not pose a noncancer risk. However, the HQ via consumption of fish brain was much higher than consumption via water and whole fish. In addition, it needs to be noted that only the concentrations of BDE-47, BDE-99, and BDE-153 were used to calculate the health risk concerning noncancer endpoints, which might slightly underestimate the HQ values of PBDEs. 

#### 3.5.2. Ecotoxicity Assessment

**Figure 6:**
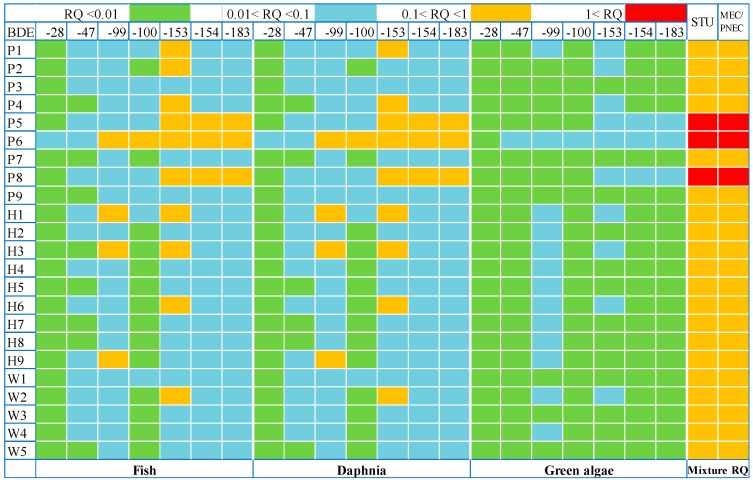
Risk quotients (RQs) of PBDEs for green algae, daphnia, and fish in water from the Chaohu Lake basin.

The ecotoxicity of PBDEs in the water at three trophic levels (green algae, daphnia, and fish) was investigated, using the risk quotient (RQ) approach as recommended [48]. Considering the possible joint effects of these PBDEs with a similar mode of action, on this basis, we estimated and assessed the expected joint risk of the PBDE mixtures. The environmental risks for each PBDE and their mixture were calculated based on the toxicology data, as shown in Figure 6. The values of median effect concentration (EC50) or median lethal concentration (LC50) (toxicology data for fish, daphnia, and green algae) for PNEC calculations are provided in Table S13, as in Xiong et al. [49]. To better elucidate the risk levels, the RQs were classified into four risk levels: RQ < 0.01, non-risk; 0.01 ≤ RQ < 0.1, low risk; 0.1 ≤ RQ < 1, medium risk; 1 ≤ RQ, high risk to aquatic organisms.

As shown in Figure 6, the RQs of the eight PBDEs detected in the water were below 1 at all the sampling sites. Overall, a large majority of RQ values for green algae were below 0.01, indicating little risk of PBDEs to the relevant sensitive aquatic organisms. Regarding fish and daphnia in the 23 sampling sites, 26.1% of RQs were less than 0.01, 62.2% of RQs were in the range 0.01–0.1, and 13.7% of RQs were in the range 0.1–1 for seven PBDEs (excluding BDE-209), in which the RQs of BDE-153 accounted for 47.6%, followed by BDE-99, BDE-154, and BDE-183. In more than half of the sampling sites, the RQ of at least one PBDE showed medium risk. Though the RQ value of each PBDE in all the sampling sites is less than 1, the MRQs based on Toxic Unit (TU) analyses and the sum MEC/PNEC exceeded the alert value in sampling sites P5, P6, and P8, where effects on aquatic organisms would be expected. Furthermore, the MRQ at other sampling sites (excluding P5, P6, and P8) was higher than 0.1, suggesting medium risk to the aquatic organisms. Summing up the TU trophic level by trophic level in order to calculate an MRQ_STU_ follows the conceptual idea of concentration addition more closely than the sum of MEC/PNEC, which yields the MRQ_MEC/PNEC_ (Table S14). The ratio between MRQ_STU_ and MRQ_MEC/PNEC_ never exceeds 1.1 in all the 23 sampling sites. This is because the PBDEs dominating the mixtures have a quite similar ecotoxicological profile, with daphnia consistently being the most sensitive trophic level. A similar result was observed in some STP effluents and receiving water of effluent [50,51]. In the case of very different ecotoxicological profiles of the dominating mixture components, the ratio of MRQ_STU_ and MRQ_MEC/PNCE_ could theoretically reach 3 [48]. It should be pointed out that BDE-209 congener has a number of additional analytical difficulties, and the concentration in the water was below the detection limits. If the undetectable values were replaced with 1/2 MDL, the RQs of BDE-209 in all the sampling sites for sensitive aquatic organisms exceed 1.

## 4. Conclusions

The present research work has provided valuable information on the PBDE contamination level in water, surface sediments, and biota samples collected from the Chaohu Lake basin, China, with average concentrations of 0.11–4.48 ng/L, 0.06–5.41 ng/g (dw), and 0.02–1.50 ng/g (dw), respectively. Temporal variation in surface water revealed higher pollutant levels in the autumn (October). Spatial variation of PBDEs in surface water showed that relatively high concentrations of PBDEs were detected in the western part of Chaohu Lake, and inflowing rivers are primary sources of PBDEs in Chaohu Lake. The levels of PBDEs in STP sludge samples were higher than those detected in the surface sediment. Tissue distribution of PBDEs in fish was also studied, indicating that the brain is one of the major targeted storage sites for PBDEs in crucian carp. The PBDE homologue distribution patterns in the three environmental media showed that Penta-BDE (BDE-47, BDE-99, and BDE-153) were the major PBDE congeners in the water and biological samples, while PBDE composition in the sediment/sludge was predominated by Deca-BDE (BDE-209). The noncancer risks of PBDEs were all below 10^−5^; hence, the water and fish quality of Chaohu Lake was considered safe for humans based on the concentrations of PBDEs. The RQ indicates that PBDEs in P5, P6, and P8 water samples posed a high mixture ecological risk for the aquatic organisms (algae, daphnias, and/or fish). More attention should be paid to this aquatic environment.

## Supplementary Materials

Supplementary data to this article can be found online at http://www.mdpi.com/1660-4601/15/7/1529/s1. Table S1: Optimized MS/MS parameters of the eight PBDEs, Table S2: Recovery rates of the eight PBDEs in water samples. Table S3: Recovery rates of the eight PBDEs in sediment/biota samples, Table S4: Method detection limits (MDLs) for the eight PBDEs, Table S5: Regression equations, correlation coefficients, and retention times of the eight PBDEs, Table S6: Contents of PBDEs in main rivers connected to Chaohu Lake (ng/L), Table S7: Contents of PBDEs in the effluents of main sewage treatment plants surrounding Chaohu Lake (ng/L), Table S8: Contents of PBDEs in water from Chaohu Lake (ng/L), Table S9: Contents of PBDEs in sediments of rivers flowing into Chaohu Lake and the sludge of sewage treatment plants (ng/g), Table S10: Contents of PBDEs in sediments from Chaohu Lake (ng/g), Table S11: Concentrations of PBDEs in aquatic species from Chaohu Lake (ng/g), Table S12: Estimated daily intake (EDI) and Hazard quotient (HQ) for BDE-47, BDE-99, and BDE-153, Table S13: The values of PNEC for fish, daphnia, and green algae, Table S14: The base set of acute toxicity for the calculation of MRQ_MEC/PNEC_ and MRQ_STU_.
