# Supplementary material for: Polybrominated Diphenyl Ethers (PBDEs) in a Large, Highly Polluted Freshwater Lake, China: Occurrence, Fate, and Risk Assessment
Source: Int J Environ Res Public Health. 2018 Jul 19;15(7):1529. doi: 10.3390/ijerph15071529 (PMC6068772; doi:10.3390/ijerph15071529)
Supplement: Supplementary file 1 [file ijerph-15-01529-s001.pdf]

Polybrominated Diphenyl Ethers (PBDEs) in a Large, Highly Polluted Freshwater Lake, China: Occurrence, Fate, and Risk Assessment

Jianchao Liu [1], Guanghua Lu [1,2,*], Fuhai Zhang [3,*], Matthew Nkoom [1], Zhenhua Yan [1] and Donghai Wu [1]

[1] Key Laboratory of Integrated Regulation and Resources Development, College of Environment, Hohai University, Nanjing 210098, China; jianchao-liu@hhu.edu.cn
[2] College of Hydraulic and Civil Engineering, XiZang Agricultural and Animal Husbandry College, Linzhi, China
[3] Anhui Environmental Monitoring Center, Hefei 230061, China
* Correspondence: ghlu@hhu.edu.cn (G.L.); zfh168@163.com (F.Z.)

Supplementary Information

Table S1. Optimized MS/MS parameters of the eight PBDEs.
Table S2. Recovery rates of the eight PBDEs in water samples.
Table S3. Recover y rates of the eight PBDEs in sediment/biota samples.
Table S4. Method detection limits (MDLs) for the eight PBDEs.
Table S5. Regression equations, correlation coefficients and retention time of the eight PBDEs.
Table S6. Contents of PBDEs in main rivers connected to Chaohu Lake (ng/L).
Table S7. Contents of PBDEs in the effluents of main sewage treatment plants surrounding Chaohu Lake (ng/L).
Table S8. Contents of PBDEs in water from Chaohu Lake (ng/L).
Table S9. Contents of PBDEs in sediments of rivers flowing into Chaohu Lake and the sludge of sewage treatment plants (ng/g).
Table S10. Contents of PBDEs in sediments from Chaohu Lake (ng/g).
Table S11. Concentrations of PBDEs in aquatic species from Chaohu Lake (ng/g).
Table S12. Estimated daily intake (EDI) and Hazard quotient (HQ) for BDE-47, BDE-99 and BDE-153.
Table S13. The values of PNEC for fish, daphnia and green algae.
Table S14. The base set of acute toxicity for the calculation of $MRQ_{MEC/PNEC}$ and MRQ_{STU}.

Materials and Methods

Sample Extraction and Analyses

Water samples were filtered through 0.45 μm glass fiber filters (GF/F, Whatman, Clifton, NJ, USA) to remove particles. The water samples filtrate mixed with 0.1 volumes of methanol and sonicated for 5 min. Then the filtrate was extracted by solid phase extraction (SPE) on HLB cartridges (200 mg, Waters, Massachusetts, USA). The cartridges were preconditioned with 5 mL dichlormethane, 5 mL ethyl acetate, 5 mL methanol and 5 mL water. The samples were eluted from the cartridges using 3 mL dichlormethane and 3 mL ethyl acetate. The extracts were evaporated to dryness under a stream of nitrogen, reconstituted with 2 mL n-hexane.

Fish tissue (brain, liver, gills and muscle) samples were homogenized and weighed prior to extracting with an accelerated solvent extractor followed by an ASE 300 (Dionex Ltd., Sunnyvale, CA, USA). Weighed samples were mixed with 5 g of anhydrous sodium sulfate and

were loaded into 33 mL ASE cells on top of 10 g of activated Florisil, which was used as a clean-up. Cells were spiked with 10 μL of 1 ng/μL $^{13}C_{12}$-labelled 2,2',4,4',5,5'-hexa-BDE (BDE-153) as a recovery internal standard (RIS) solution. The fish sample was then extracted at 100°C with a mixture of n-hexane/dichloromethane (DCM) (4:1, v/v) working at a constant pressure of 1500 psi, a flush volume of 60% and a purge time of 90 s. Two static extraction cycles of 5 min each were applied to achieve the maximum recovery of the analytes. After extraction, the solvents were evaporated down to 1.0 mL and analyzed by GC/MS/MS.

PBDEs and bromophenols were analyzed on a Bruker450 GC-320 triple quadrupole mass spectrometer (Fremont, CA, USA) fitted with a DB-5HT capillary column (15 cm × 250 μm × 0.1 μm), combined with the multiple-reaction monitor (MRM) mode of tandem mass spectrometry. The mass spectrometer was operated in negative electrospray ionization mode (ESI-) using MRM. The quantification of PBDEs was conducted using $^{13}C_{12}$-labelled BDE-153 as an internal standards.

A total of 39 PBDEs were analyzed by GC-MS/MS (EI, 70 eV). The GC-MS/MS was operated in pulsed splitless injection mode with an injector temperature of 340℃.The helium carrier gas flow rate was 1.3 mL/min, and the oven temperature program was as follows: 40°C, 230°C (20°C/min), 285°C (6°C/min), 340°C (25°C/min), held for 7 min. The interface, source and quadrupole temperature were set at 300, 300 and 40°C, respectively, and the MRM parameters are listed in Table S1.

Parameter Measurement and Statistical Analysis

Health Risk Assessment

The estimated daily intake (EDI, ng/kg-bw/day) of PBDEs by adult was calculated as follows:

$$EDI_{water} = C \times V / BW \quad (1)$$

where C is the concentration of a PBDE congener in water (ng/L); V (mL/day) is the consumption rate of water (L); and BW (kg) is the adult body weight.

$$EDI_{water} = C \times V / BW \quad (2)$$

$$EDI_{fish} = C \bullet R / BW \quad (3)$$

where C is the concentration of a PBDE congener in fish (ng/g); R is the consumption rate of fish (g/day); and BW (kg) is the adult body weight.

To assess health risks associated with exposure to PBDEs concerning non-carcinogenic endpoints, hazard quotient (HQ) values were estimated and used. It can be calculated simply by dividing the estimated daily intake by the reference dose (RfD) of PBDEs reported by the Integrated Risk Information System (IRIS) of the USEPA as follows:

$$HQ = f \bullet EDI / RfD \quad (4)$$

where f is the transfer factor of unit (which is 0.001 in this case)

According to the risk addition method, the total HQs of all PBDE congeners can be treated as the mathematical sum of the HQ values of single PBDE congener:

$$THQ = \Sigma HQ_i \quad (5)$$

Eco-toxicity assessment

Eco-toxicity of target compounds in water was assessed using the risk quotient (RQ) on non-target organisms. At three trophic levels, LC50 or EC50 for fish, daphnia and green algae associated with PBDEs were used for RQ calculation as Eq (6).

$$RQ = \frac{MEC}{PNEC} = \frac{MEC}{EC50 \text{ or } LC50/f} \quad (6)$$

where PNEC is the predicted no effect concentration (mg/L),estimated as a quotient of toxicological relevant concentration (EC50 or LC50) with a security factor (f = 1000). The values of EC50 or LC50 for fish, daphnia and green algae for PNEC calculation were provided in the Table S13.

Two approaches for calculating the mixture risk quotient (MRQ) are outlined by Eq. (7): the calculation of MRQ, based on the sum of MEC/PNEC values ($MRQ_{MEC/PNEC}$); and Eq. (8): the calculation of MRQ, based on the sum of toxic units (STUs) for the most sensitive trophic level (MRQ_{STU}). The acute toxicity data EC50 and LC50 are represented by EC50 in Eqs. (7) and (8).

$$MRQ_{MEC/PNEC} = \sum_{i=1}^{n} \frac{MEC_{ij}}{PNEC_i} = \sum_{i}^{n} \frac{MEC_{ij}}{\min(EC50_{aglae}, EC50_{daphnids}, EC50_{fish})_i \times (1/AF_i)} \quad (7)$$

$$MRQ_{STU} = \max(STU_{aglae}, STU_{daphnids}, STU_{fish}) \times AF = \max\left(\sum_{i=1}^{n} \frac{MEC_{ij}}{EC50_{i,aglae}}, \sum_{i}^{n} \frac{MEC_{ij}}{EC50_{i,daphnids}}, \sum_{i}^{n} \frac{MEC_{ij}}{EC50_{i,fish}}\right) \times AF \quad (8)$$

where TU and STU are the "toxic unit (MEC/EC50)" and the "sum of toxic unit", respectively.

Table S1. Optimized MS/MS parameters of the eight PBDEs.

Compounds	Retention time (min)	Precursor ion (m/z)	Product ion (m/z)	Collision energy (eV)
BDE-28	7.5-9.0	407.8(M+2)	247.9	25
BDE-47	9.0-10.0	485.7(M)	325.9	25
BDE-99<br>BDE-100	10.0-11.1	565.7(M+2)	405.9	25
BDE-153	11.1-12.3	483.9(M-Br_2)	376.9	25
BDE-154		643.6(M)	483.9	30
$^{13}C_{12}$-BDE-153	11.1-12.3	495.6	388.9	25
		655.7	495.8	30
BDE-183	12.3-14.2	721.6(M-Br)	561.6	35
BDE-209	14.2-20	800(M-Br_2)	642	40
		960(M)	802	45

Table S2. Recovery rates of the eight PBDEs in water samples.

Compounds	Water samples					
	Low concentration (ng/L)	Recovery rates (%)	Median concentration (ng/L)	Recovery rates (%)	High concentration (ng/L)	Recovery rates (%)
BDE-28		90～110		90～110		90～110
BDE-47		90～110		90～110		90～110
BDE-99		90～100		90～100		90～100
BDE-100	5.0	90～100	20	90～100	50	90～100
BDE-153		75～95		75～100		75～110
BDE-154		75～100		75～110		80～110
BDE-183		75～100		79～110		80～110
BDE-209	50	50～80	200	55～70	500	65～70

Table S3. Recover y rates of the eight PBDEs in sediment/biota samples.

Compounds	Sediment/Biota samples					
	Low concentration (ng/g)	Recovery rates (%)	Median concentration (ng/g)	Recovery rates (%)	High concentration (ng/g)	Recovery rates (%)
BDE-28		70～75		65～90		75～90
BDE-47		85～105		85～110		90～110
BDE-99		85～103		85～107		90～110
BDE-100	1.0	80～110	5	80～105	20	90～105
BDE-153		75～95		75～100		85～110
BDE-154		75～100		75～110		85～110
BDE-183		65～100		70～110		80～110
BDE-209	10	65～110	50	70～105	200	75～105

Table S4. Method detection limits (MDLs) for the eight PBDEs.

Compounds	Instrument Detection Limit （IDL, pg）	Method detection limits （MDLs）		
		Water samples (ng/L)		Sediment/biota (pg/g)
		This method	USEPA 1614A	
BDE-28	0.03	0.014	0.009	5
BDE-47	0.02	0.025	0.048	4
BDE-99	0.015	0.024	0.033	4
BDE-100	0.03	0.018	0.007	4
BDE-153	0.03	0.027	0.011	5
BDE-154	0.04	0.036	0.005	6
BDE-183	0.02	0.02	0.018	4
BDE-209	0.15	0.6	1.5	20

Table S5. Regression equations, correlation coefficients and retention time of the eight PBDEs.

Compounds	Regression equation	Correlation coefficient	Retention time （min）
BDE-28	y= 0.611x-0.921	0.997	8.100
BDE-47	y= 0.626x-0.648	0.997	9.426
BDE-100	y= 0.597x-0.033	0.997	10.392
BDE-99	y= 0.346x-0.501	0.998	10.676
BDE-154	y= 0.185x+0.081	0.9991	11.424
BDE-153	y=0.105x-0.057	0.9994	11.767
BDE-183	y= 0.232x-0.143	0.998	12.674
BDE-209	y= 0.010x-0.343	0.9996	17.030

Table S6. Contents of PBDEs in main rivers connected to Chaohu Lake (ng/L).

Compounds	Time	P1	P2	P3	P4	P5	P6	P7	P8	P9
BDE-28	June	0.16	0.23	ND	ND	ND	0.34	ND	0.15	ND
	October	0.20	ND	0.24	ND	0.23	1.36	ND	0.26	0.14
BDE-47	June	0.38	0.13	0.16	0.25	0.19	0.54	0.10	0.25	0.23
	October	0.45	0.21	0.25	0.17	0.27	1.31	0.06	0.32	0.18
BDE-99	June	0.15	0.24	0.16	0.20	0.15	0.32	0.05	0.24	0.12
	October	0.30	0.14	0.13	0.06	0.15	0.65	0.05	0.21	0.08
BDE-100	June	ND	0.17	0.06	ND	ND	0.34	ND	ND	ND
	October	0.12	ND	0.12	0.09	0.17	0.66	ND	0.22	0.12
BDE-153	June	0.06	0.21	0.05	ND	ND	0.16	ND	ND	ND
	October	0.16	0.07	ND	0.08	0.10	0.21	ND	0.11	ND
BDE-154	June	ND	ND	ND	ND	ND	ND	ND	ND	ND
	October	ND	ND	ND	ND	0.10	0.18	ND	0.11	ND
BDE-183	June	ND	ND	ND	ND	ND	ND	ND	ND	ND
	October	ND	ND	ND	ND	0.10	0.11	ND	0.09	ND
BDE-209	June	ND	ND	ND	ND	ND	ND	ND	ND	ND
	October	ND	ND	ND	ND	ND	ND	ND	ND	ND
ΣPBDEs	June	0.75	0.98	0.43	0.45	0.65	1.70	0.15	0.64	0.40

	October	1.23	0.42	0.74	0.40	1.12	4.48	0.11	1.32	0.52

ND: below the Method detection limit.

Table S7. Contents of PBDEs in the effluents of main sewage treatment plants surrounding Chaohu Lake (ng/L).

Compounds	W1	W2	W3	W4	W5
BDE-28	ND	ND	ND	ND	ND
BDE-47	0.31	0.26	0.21	0.38	ND
BDE-99	0.22	0.32	0.23	0.26	0.20
BDE-100	0.16	ND	ND	ND	ND
BDE-153	ND	0.23	ND	ND	ND
BDE-154	ND	ND	ND	ND	ND
BDE-183	ND	ND	ND	ND	ND
BDE-209	ND	ND	ND	ND	ND
ΣPBDEs	0.69	0.71	0.44	0.64	0.20

ND: below the Method detection limit.

Table S8. Contents of PBDEs in water from Chaohu Lake (ng/L).

Compounds	Time	H1	H2	H3	H4	H5	H6	H7	H8	H9
BDE-28	June	0.10	ND	ND	ND	ND	ND	ND	ND	ND
	October	0.16	ND	ND	ND	ND	ND	ND	ND	ND
BDE-47	June	0.35	0.30	0.12	0.10	ND	0.24	ND	ND	0.19
	October	0.54	0.36	0.20	0.28	ND	0.44	ND	ND	0.46
BDE-99	June	0.43	0.24	0.21	0.17	0.16	0.23	0.20	0.23	0.40
	October	0.48	0.32	0.43	0.36	0.28	0.39	0.27	0.28	0.45
BDE-100	June	ND	ND	ND	ND	ND	ND	ND	ND	ND
	October	0.15	ND	ND	ND	ND	ND	ND	ND	ND
BDE-153	June	0.14	0.10	0.12	ND	ND	0.11	ND	ND	ND
	October	0.23	ND	0.31	ND	ND	0.26	ND	ND	ND
BDE-154	June	ND	ND	ND	ND	ND	ND	ND	ND	ND
	October	ND	ND	ND	ND	ND	ND	ND	ND	ND
BDE-183	June	ND	ND	ND	ND	ND	ND	ND	ND	ND
	October	ND	ND	ND	ND	ND	ND	ND	ND	ND
BDE-209	June	ND	ND	ND	ND	ND	ND	ND	ND	ND
	October	ND	ND	ND	ND	ND	ND	ND	ND	ND
ΣPBDEs	June	1.02	0.64	0.45	0.27	0.16	0.58	0.20	0.23	0.59
	October	1.56	0.68	0.94	0.64	0.28	1.09	0.27	0.28	0.91

ND: below the Method detection limit.

Table S9. Contents of PBDEs in sediments of rivers flowing into Chaohu Lake and the sludge of sewage treatment plants (ng/g).

Compounds	P1	P2	P3	P3-1	P4	P5	W1
BDE-28	ND	ND	ND	ND	ND	ND	ND
BDE-47	0.017	0.022	0.023	0.025	0.023	0.022	0.324
BDE-99	0.023	ND	ND	ND	ND	ND	0.251
BDE-100	ND	ND	ND	ND	ND	ND	0.014
BDE-153	0.012	ND	0.014	0.013	0.013	0.014	0.294
BDE-154	ND	ND	0.021	0.025	ND	0.021	0.047
BDE-183	0.056	0.020	0.024	0.022	0.020	0.021	0.081
BDE-209	1.27	0.27	1.45	3.96	0.20	1.34	10.5
ΣPBDEs	1.38	0.312	1.53	4.05	0.256	1.42	11.5

ND: below the Method detection limit.

Table S10. Contents of PBDEs in sediments from Chaohu Lake (ng/g).

Compounds	BDE-28	BDE-47	BDE-99	BDE-100	BDE-153	BDE-154	BDE-183	BDE-209	ΣPBDEs
H1	0.032	0.043	0.036	0.16	0.033	0.071	0.086	4.95	5.41
H2	ND	0.032	ND	ND	0.023	0.039	ND	2.48	2.57
H3	ND	0.021	0.026	ND	0.014	ND	ND	1.21	1.27
H4	ND	ND	0.01	0.06	ND	ND	0.04	1.80	1.91
H5	ND	0.025	0.030	ND	ND	ND	ND	0.55	0.605
H6	ND	0.025	ND	ND	0.013	0.019	0.019	0.54	0.616
H7	ND	0.021	0.030	ND	0.013	0.019	ND	0.45	0.533
H8	ND	0.026	0.032	ND	ND	ND	ND	ND	0.058
H9	ND	0.027	0.035	ND	0.013	ND	ND	ND	0.075
H10	ND	0.042	ND	ND	0.018	0.022	0.022	4.56	4.66
H11	ND	0.015	0.014	0.051	0.024	0.030	0.047	2.71	2.89
H12	ND	0.026	0.015	0.067	0.017	0.019	0.033	1.54	1.72
H13	ND	0.021	0.013	ND	0.012	0.019	ND	ND	0.065
H14	ND	0.022	0.024	ND	0.012	ND	ND	ND	0.058

ND: below the Method detection limit.

Table S11. Concentrations of PBDEs in aquatic species from Chaohu Lake (ng/g).

**Species**	**BDE-28**	**BDE-47**	**BDE-99**	**BDE-100**	**BDE-153**	**BDE-154**	**BDE-183**	**BDE-209**	**ΣPBDEs**
River shrimp[a]	ND	ND	0.05	ND	ND	ND	ND	ND	0.05
Chinese hooksnout carp[a]	ND	0.115	0.079	0.056	0.113	0.126	0.035	ND	0.524
Crucian carp[a]	ND	0.021	ND	ND	ND	ND	ND	ND	0.021
Topmouth culter[a]	0.008	0.013	ND	ND	ND	ND	ND	ND	0.021
Silver fish[a]	ND	0.026	0.012	ND	ND	0.012	ND	ND	0.05
Chinese hooksnout carp [b]	ND	ND	0.014	ND	0.008	0.021	ND	ND	0.043
Silver fish[b]	ND	ND	0.009	ND	0.011	0.029	ND	ND	0.049
Topmouth culter[b]	ND	0.018	ND	ND	0.026	0.011	0.009	ND	0.064
Common carp[b]	ND	0.024	ND	ND	0.013	0.018	ND	ND	0.275
Crucian carp (Brain)[b]	ND	0.549	ND	ND	1.25	0.028	ND	ND	1.876
Crucian carp (Gill)[b]	ND	0.066	ND	ND	0.047	ND	ND	ND	0.205
Crucian carp (Liver)[b]	ND	0.025	0.034	ND	0.032	ND	ND	ND	0.091
Crucian carp (Intestines)[b]	ND	0.018	0.022	ND	ND	ND	ND	ND	0.04

[a]: Biota samples were collected from S1 site; [b]: Biota samples were collected from S2 site; ND: below the Method detection limit.

Table S12. Estimated daily intake (EDI) and Hazard quotient (HQ) for BDE-47, BDE-99 and BDE-153.

Sites/Biology	EDI(ng/kg/day)			Sum of EDI	HQ			Sum of HQ
	BDE-47	BDE-99	BDE-153		BDE-47	BDE-99	BDE-153	
P1	1.6E-02	1.0E-02	5.6E-03	3.2E-02	1.6E-06	1.0E-06	2.8E-07	2.9E-06
P2	7.3E-03	4.9E-03	2.4E-03	1.5E-02	7.3E-07	4.9E-07	1.2E-07	1.3E-06
P3	8.7E-03	4.5E-03	4.5E-04	1.4E-02	8.7E-07	4.5E-07	2.3E-08	1.3E-06
P4	5.9E-03	2.1E-03	2.8E-03	1.1E-02	5.9E-07	2.1E-07	1.4E-07	9.4E-07
P5	9.4E-03	5.2E-03	3.5E-03	1.8E-02	9.4E-07	5.2E-07	1.7E-07	1.6E-06
P6	4.6E-02	2.3E-02	7.3E-03	7.6E-02	4.6E-06	2.3E-06	3.7E-07	7.2E-06
P7	2.1E-03	1.7E-03	4.5E-04	4.3E-03	2.1E-07	1.7E-07	2.3E-08	4.1E-07
P8	1.1E-02	7.3E-03	3.8E-03	2.2E-02	1.1E-06	7.3E-07	1.9E-07	2.0E-06
P9	6.3E-03	2.8E-03	4.5E-04	9.5E-03	6.3E-07	2.8E-07	2.3E-08	9.3E-07
H1	1.9E-02	1.7E-02	8.0E-03	4.4E-02	1.9E-06	1.7E-06	4.0E-07	4.0E-06
H2	1.3E-02	1.1E-02	4.5E-04	2.4E-02	1.3E-06	1.1E-06	2.3E-08	2.4E-06
H3	7.0E-03	1.5E-02	1.1E-02	3.3E-02	7.0E-07	1.5E-06	5.4E-07	2.7E-06
H4	9.8E-03	1.3E-02	4.5E-04	2.3E-02	9.8E-07	1.3E-06	2.3E-08	2.3E-06
H5	4.2E-04	9.8E-03	4.5E-04	1.1E-02	4.2E-08	9.8E-07	2.3E-08	1.0E-06
H6	1.5E-02	1.4E-02	9.1E-03	3.8E-02	1.5E-06	1.4E-06	4.5E-07	3.3E-06

H7	4.2E-04	9.4E-03	4.5E-04	1.0E-02	4.2E-08	9.4E-07	2.3E-08	1.0E-06
H8	4.2E-04	9.8E-03	4.5E-04	1.1E-02	4.2E-08	9.8E-07	2.3E-08	1.0E-06
H9	1.6E-02	1.6E-02	4.5E-04	3.2E-02	1.6E-06	1.6E-06	2.3E-08	3.2E-06
River shrimp	3.3E-03	8.4E-02	4.2E-03	9.1E-02	3.3E-07	8.4E-06	2.1E-07	8.9E-06
Chinese hooksnout carp	1.9E-01	1.3E-01	1.9E-01	5.1E-01	1.9E-05	1.3E-05	9.5E-06	4.2E-05
Crucian carp	3.5E-02	3.3E-03	4.2E-03	4.3E-02	3.5E-06	3.3E-07	2.1E-07	4.1E-06
Silver fish	4.4E-02	2.0E-02	1.8E-02	8.2E-02	4.4E-06	2.0E-06	9.2E-07	7.3E-06
Topmouth culter	3.0E-02	3.3E-03	4.4E-02	7.7E-02	3.0E-06	3.3E-07	2.2E-06	5.5E-06
Common carp	4.0E-02	3.7E-01	2.2E-02	4.3E-01	4.0E-06	3.7E-05	1.1E-06	4.2E-05
Crucian carp (Brain)	1.1E+00	1.0E-01	2.6E+00	3.9E+00	1.1E-04	1.0E-05	1.3E-04	2.6E-04

Table S13. The values of PNEC for fish, daphnia and green algae.

PNEC	fish (ng/L)	daphnia (ng/L)	green algae (ng/L)
BDE-28	110	89	292
BDE-47	21	18	84
BDE-100	4	4	24
BDE-99	4	4	24
BDE-154	0.693	0.721	7
BDE-153	0.693	0.721	7
BDE-183	0.124	0.139	1.78
BDE-209	0.000656	0.000947	0.0335

Table S14. The base set of acute toxicity for the calculation of $MRQ_{MEC/PNEC}$ and MRQ_{STU}.

**Sites**	**Toxic Units**	**BDE-28**	**BDE-47**	**BDE-99**	**BDE-100**	**BDE-153**	**BDE-154**	**BDE-183**	**Sum**
P1	$TU_{algae} \times 10^{-3}$	0.68	5.36	12.50	5.00	22.86	2.57	5.62	0.05
	$TU_{daphnids} \times 10^{-3}$	2.25	25.00	75.00	30.00	221.91	24.97	71.94	0.45
	$TU_{fish} \times 10^{-3}$	1.82	21.43	75.00	30.00	230.88	25.97	80.65	0.47
	MEC/min(EC50) $\times 10^{-3}$	2.25	25.00	75.00	30.00	230.88	25.97	80.65	0.47
P2	$TU_{algae} \times 10^{-3}$	0.02	2.50	5.83	0.38	10.00	2.57	5.62	0.03
	$TU_{daphnids} \times 10^{-3}$	0.08	11.67	35.00	2.25	97.09	24.97	71.94	0.24
	$TU_{fish} \times 10^{-3}$	0.06	10.00	35.00	2.25	101.01	25.97	80.65	0.25
	MEC/min(EC50) $\times 10^{-3}$	0.08	11.67	35.00	2.25	101.01	25.97	80.65	0.26
P3	$TU_{algae} \times 10^{-3}$	0.82	2.98	5.42	5.00	1.86	2.57	5.62	0.02
	$TU_{daphnids} \times 10^{-3}$	2.70	13.89	32.50	30.00	18.03	24.97	71.94	0.19
	$TU_{fish} \times 10^{-3}$	2.18	11.90	32.50	30.00	18.76	25.97	80.65	0.20
	MEC/min(EC50) $\times 10^{-3}$	2.70	13.89	32.50	30.00	18.76	25.97	80.65	0.20
P4	$TU_{algae} \times 10^{-3}$	0.02	2.02	2.50	3.75	11.43	2.57	5.62	0.03
	$TU_{daphnids} \times 10^{-3}$	0.08	9.44	15.00	22.50	110.96	24.97	71.94	0.25
	$TU_{fish} \times 10^{-3}$	0.06	8.10	15.00	22.50	115.44	25.97	80.65	0.27
	MEC/min(EC50) $\times 10^{-3}$	0.08	9.44	15.00	22.50	115.44	25.97	80.65	0.27
P5	$TU_{algae} \times 10^{-3}$	0.79	3.21	6.25	7.08	14.29	14.29	56.18	0.10
	$TU_{daphnids} \times 10^{-3}$	2.58	15.00	37.50	42.50	138.70	138.70	719.42	1.09
	$TU_{fish} \times 10^{-3}$	2.09	12.86	37.50	42.50	144.30	144.30	806.45	1.19
	MEC/min(EC50) $\times 10^{-3}$	2.58	15.00	37.50	42.50	144.30	144.30	806.45	1.19
P6	$TU_{algae} \times 10^{-3}$	4.66	15.60	27.08	27.50	30.00	25.71	61.80	0.19
	$TU_{daphnids} \times 10^{-3}$	15.28	72.78	162.50	165.00	291.26	249.65	791.37	1.75
	$TU_{fish} \times 10^{-3}$	12.36	62.38	162.50	165.00	303.03	259.74	887.10	1.85
	MEC/min(EC50) $\times 10^{-3}$	15.28	72.78	162.50	165.00	303.03	259.74	887.10	1.87
P7	$TU_{algae} \times 10^{-3}$	0.02	0.71	2.08	0.38	1.86	2.57	5.62	0.01
	$TU_{daphnids} \times 10^{-3}$	0.08	3.33	12.50	2.25	18.03	24.97	71.94	0.13
	$TU_{fish} \times 10^{-3}$	0.06	2.86	12.50	2.25	18.76	25.97	80.65	0.14
	MEC/min(EC50) $\times 10^{-3}$	0.08	3.33	12.50	2.25	18.76	25.97	80.65	0.14
P8	$TU_{algae} \times 10^{-3}$	0.89	3.81	8.75	9.17	15.71	15.71	50.56	0.10
	$TU_{daphnids} \times 10^{-3}$	2.92	17.78	52.50	55.00	152.57	152.57	647.48	1.08
	$TU_{fish} \times 10^{-3}$	2.36	15.24	52.50	55.00	158.73	158.73	725.81	1.17
	MEC/min(EC50) $\times 10^{-3}$	2.92	17.78	52.50	55.00	158.73	158.73	725.81	1.17
P9	$TU_{algae} \times 10^{-3}$	0.48	2.14	3.33	5.00	1.86	2.57	5.62	0.02
	$TU_{daphnids} \times 10^{-3}$	1.57	10.00	20.00	30.00	18.03	24.97	71.94	0.18
	$TU_{fish} \times 10^{-3}$	1.27	8.57	20.00	30.00	18.76	25.97	80.65	0.19
	MEC/min(EC50) $\times 10^{-3}$	1.57	10.00	20.00	30.00	18.76	25.97	80.65	0.19

H1	$TU_{algae} \times 10^{-3}$	0.55	6.43	20.00	6.25	32.86	2.57	5.62	0.07
	$TU_{daphnids} \times 10^{-3}$	1.80	30.00	120.00	37.50	319.00	24.97	71.94	0.61
	$TU_{fish} \times 10^{-3}$	1.45	25.71	120.00	37.50	331.89	25.97	80.65	0.62
	MEC/min(EC50) $\times 10^{-3}$	1.80	30.00	120.00	37.50	331.89	25.97	80.65	0.63
H2	$TU_{algae} \times 10^{-3}$	0.02	4.29	13.33	0.38	1.86	2.57	5.62	0.03
	$TU_{daphnids} \times 10^{-3}$	0.08	20.00	80.00	2.25	18.03	24.97	71.94	0.22
	$TU_{fish} \times 10^{-3}$	0.06	17.14	80.00	2.25	18.76	25.97	80.65	0.22
	MEC/min(EC50) $\times 10^{-3}$	0.08	20.00	80.00	2.25	18.76	25.97	80.65	0.23
H3	$TU_{algae} \times 10^{-3}$	0.02	2.38	17.92	0.38	44.29	2.57	5.62	0.07
	$TU_{daphnids} \times 10^{-3}$	0.08	11.11	107.50	2.25	429.96	24.97	71.94	0.65
	$TU_{fish} \times 10^{-3}$	0.06	9.52	107.50	2.25	447.33	25.97	80.65	0.67
	MEC/min(EC50) $\times 10^{-3}$	0.08	11.11	107.50	2.25	447.33	25.97	80.65	0.67
H4	$TU_{algae} \times 10^{-3}$	0.02	3.33	15.00	0.38	1.86	2.57	5.62	0.03
	$TU_{daphnids} \times 10^{-3}$	0.08	15.56	90.00	2.25	18.03	24.97	71.94	0.22
	$TU_{fish} \times 10^{-3}$	0.06	13.33	90.00	2.25	18.76	25.97	80.65	0.23
	MEC/min(EC50) $\times 10^{-3}$	0.08	15.56	90.00	2.25	18.76	25.97	80.65	0.23
H5	$TU_{algae} \times 10^{-3}$	0.02	0.14	11.67	0.38	1.86	2.57	5.62	0.02
	$TU_{daphnids} \times 10^{-3}$	0.08	0.67	70.00	2.25	18.03	24.97	71.94	0.19
	$TU_{fish} \times 10^{-3}$	0.06	0.57	70.00	2.25	18.76	25.97	80.65	0.20
	MEC/min(EC50) $\times 10^{-3}$	0.08	0.67	70.00	2.25	18.76	25.97	80.65	0.20
H6	$TU_{algae} \times 10^{-3}$	0.02	5.24	16.25	0.38	37.14	2.57	5.62	0.07
	$TU_{daphnids} \times 10^{-3}$	0.08	24.44	97.50	2.25	360.61	24.97	71.94	0.58
	$TU_{fish} \times 10^{-3}$	0.06	20.95	97.50	2.25	375.18	25.97	80.65	0.60
	MEC/min(EC50) $\times 10^{-3}$	0.08	24.44	97.50	2.25	375.18	25.97	80.65	0.61
H7	$TU_{algae} \times 10^{-3}$	0.02	0.14	11.25	0.38	1.86	2.57	5.62	0.02
	$TU_{daphnids} \times 10^{-3}$	0.08	0.67	67.50	2.25	18.03	24.97	71.94	0.19
	$TU_{fish} \times 10^{-3}$	0.06	0.57	67.50	2.25	18.76	25.97	80.65	0.20
	MEC/min(EC50) $\times 10^{-3}$	0.08	0.67	67.50	2.25	18.76	25.97	80.65	0.20
H8	$TU_{algae} \times 10^{-3}$	0.02	0.14	11.67	0.38	1.86	2.57	5.62	0.02
	$TU_{daphnids} \times 10^{-3}$	0.08	0.67	70.00	2.25	18.03	24.97	71.94	0.19
	$TU_{fish} \times 10^{-3}$	0.06	0.57	70.00	2.25	18.76	25.97	80.65	0.20
	MEC/min(EC50) $\times 10^{-3}$	0.08	0.67	70.00	2.25	18.76	25.97	80.65	0.20
H9	$TU_{algae} \times 10^{-3}$	0.02	5.48	18.75	0.38	1.86	2.57	5.62	0.03
	$TU_{daphnids} \times 10^{-3}$	0.08	25.56	112.50	2.25	18.03	24.97	71.94	0.26
	$TU_{fish} \times 10^{-3}$	0.06	21.90	112.50	2.25	18.76	25.97	80.65	0.26
	MEC/min(EC50) $\times 10^{-3}$	0.08	25.56	112.50	2.25	18.76	25.97	80.65	0.27
W1	$TU_{algae} \times 10^{-3}$	0.02	3.69	9.17	6.67	1.86	2.57	5.62	0.03
	$TU_{daphnids} \times 10^{-3}$	0.08	17.22	55.00	40.00	18.03	24.97	71.94	0.23
	$TU_{fish} \times 10^{-3}$	0.06	14.76	55.00	40.00	18.76	25.97	80.65	0.24
	MEC/min(EC50) $\times 10^{-3}$	0.08	17.22	55.00	40.00	18.76	25.97	80.65	0.24
W2	$TU_{algae} \times 10^{-3}$	0.02	3.10	13.33	0.38	32.86	2.57	5.62	0.06
	$TU_{daphnids} \times 10^{-3}$	0.08	14.44	80.00	2.25	319.00	24.97	71.94	0.51
	$TU_{fish} \times 10^{-3}$	0.06	12.38	80.00	2.25	331.89	25.97	80.65	0.53
	MEC/min(EC50) $\times 10^{-3}$	0.08	14.44	80.00	2.25	331.89	25.97	80.65	0.54
W3	$TU_{algae} \times 10^{-3}$	0.02	2.50	9.58	0.38	1.86	2.57	5.62	0.02
	$TU_{daphnids} \times 10^{-3}$	0.08	11.67	57.50	2.25	18.03	24.97	71.94	0.19
	$TU_{fish} \times 10^{-3}$	0.06	10.00	57.50	2.25	18.76	25.97	80.65	0.20
	MEC/min(EC50) $\times 10^{-3}$	0.08	11.67	57.50	2.25	18.76	25.97	80.65	0.20
W4	$TU_{algae} \times 10^{-3}$	0.02	4.52	10.83	0.38	1.86	2.57	5.62	0.03
	$TU_{daphnids} \times 10^{-3}$	0.08	21.11	65.00	2.25	18.03	24.97	71.94	0.20
	$TU_{fish} \times 10^{-3}$	0.06	18.10	65.00	2.25	18.76	25.97	80.65	0.21
	MEC/min(EC50) $\times 10^{-3}$	0.08	21.11	65.00	2.25	18.76	25.97	80.65	0.21
W5	$TU_{algae} \times 10^{-3}$	0.02	0.14	8.33	0.38	1.86	2.57	5.62	0.02
	$TU_{daphnids} \times 10^{-3}$	0.08	0.67	50.00	2.25	18.03	24.97	71.94	0.17
	$TU_{fish} \times 10^{-3}$	0.06	0.57	50.00	2.25	18.76	25.97	80.65	0.18
	MEC/min(EC50) $\times 10^{-3}$	0.08	0.67	50.00	2.25	18.76	25.97	80.65	0.18
